# Supplementary material for: A host defense peptide mimetic, brilacidin, potentiates caspofungin antifungal activity against human pathogenic fungi
Source: Nat Commun. 2023 Apr 12;14:2052. doi: 10.1038/s41467-023-37573-y (PMC10090755; doi:10.1038/s41467-023-37573-y)
Supplement: Supplementary file 1 — Supplementary Files [file 41467_2023_37573_MOESM1_ESM.pdf]

Supplementary Figure S1 – BRI and GELDA display na aditive effect Against *A. fumigatus*

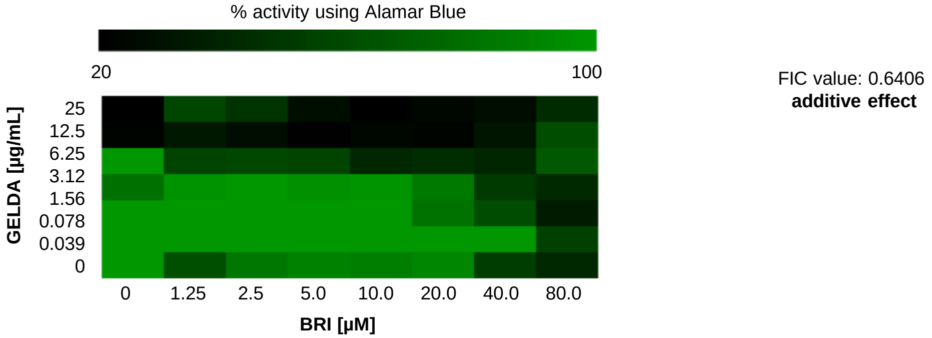

Supplementary Figure S1- The Fractional Inhibitory Concentration (FIC) index for BRI+GELDA. The heat map was generated by measurement of the metabolic activity using Alamar blue. The % of activity is based on *A. fumigatus* grown for 48 h at 37°C in the absence of BRI (ranging from 0-80µM) and GELDA (ranging from 0-25µg/mLResults show the average of three independent experiments.

Supplementary Figure S2 - Caspofungin displays a fungistatic activity against the wild-type, Δ*mpkA*, and Δ*calA* strains

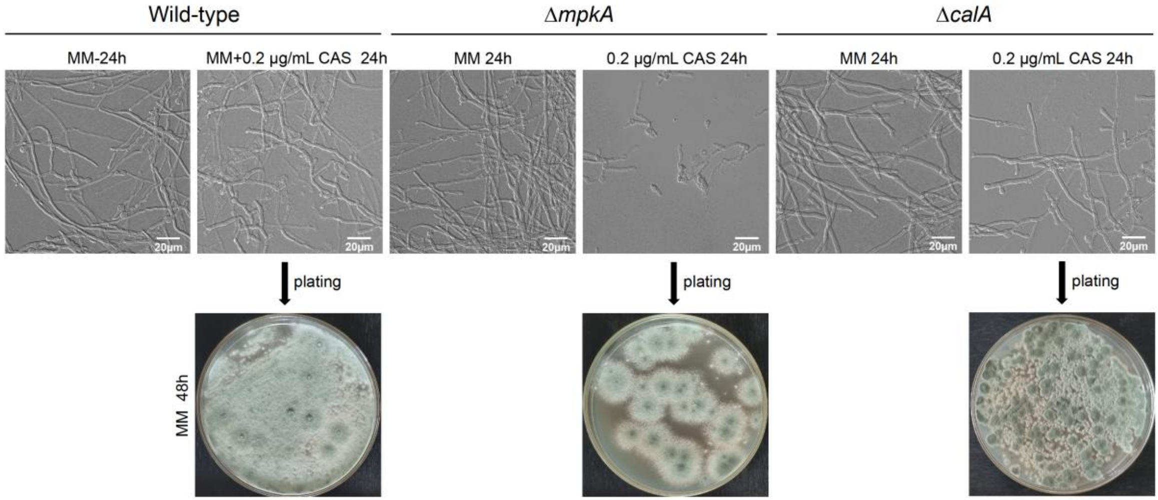

Supplementary Figure S2 – Caspofungin displays a fungistatic activity against the wild-type, Δ*mpkA*, and Δ*calA* strains. The wild-type, Δ*mpkA*, and Δ*calA* (2 x 10^3 conidia) were grown for 24 h in 200 µl liquid minimal medium (MM) supplemented or not with caspofungin 0.2 µg/ml for 24 h at 37°C. The 200 µl were plated on solid MM and Incubated for 48 hs at 37°C. The experiment was repeated independently 3 times and presented similar results.

Supplementary Figure S3 – The *A. fumigatus* chitin synthases mRNA levels after exposure to caspofungin and brilacidin.

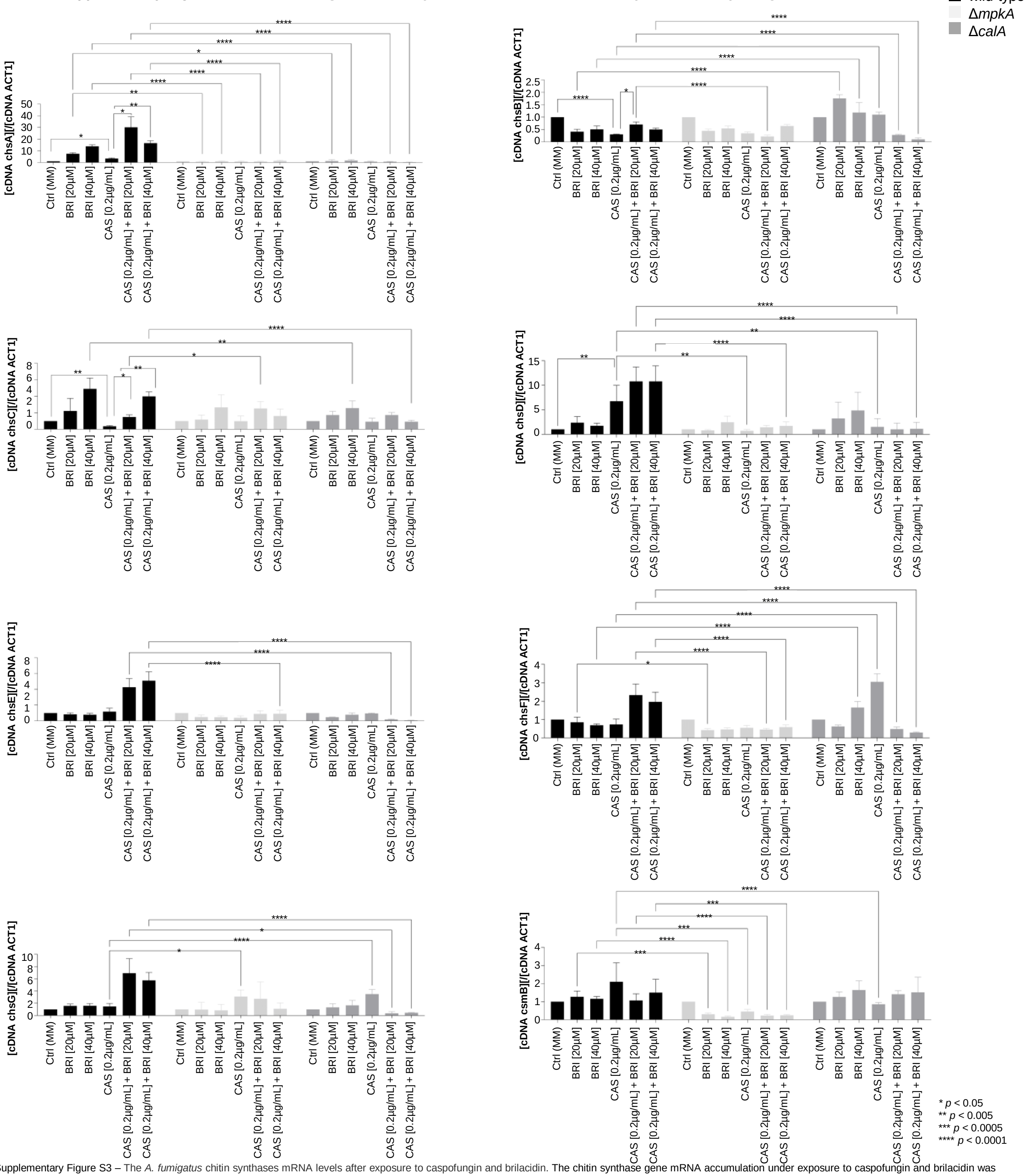

Supplementary Figure S3 – The *A. fumigatus* chitin synthases mRNA levels after exposure to caspofungin and brilacidin. The chitin synthase gene mRNA accumulation under exposure to caspofungin and brilacidin was measured in the wild-type, Δ*mpkA* and Δ*calA* strains. After 24h of growth at 37°C in MM, the mycelia were transferred to minimal medium (MM) containing (or not) CAS, BRI or combination of both drugs for 60 min. The results are expressed as the average fold increase ±SD of the control cDNA (MM without drug) for a specific gene from three independent biological experiments with 3 technical repetitions each (n=3; 2way ANOVA, Tukey´s post-test; * $p < 0.05$, ** $p < 0.005$, ***, $p < 0.0005$, and ****, $p < 0.0001$).

Supplementary Table S1 - BRI+VOR cannot overcome *A. fumigatus* VOR-resistance

				VOR [µg/ml]		BRI [µM]	0.5 µg/ml VOR		2 µg/ml VOR	
**Strain**	**Mechanism of VOR^R**	**MIC VOR [µg/ml]**	**MIC BRI [µM]**	**0.5**	**2**	**20**	**20 µM BRI**	**40 µM BRI**	**20 µM BRI**	**40 µM BRI**
**WT**	WT	2	>80	---	---	+	---	---	---	---
**CYP-15-184**	TR34/L98H	8	>80	+	+	+	+	+	+	+
**CYP-15-190**	TR34/L98H	8	>80	+	+	+	+	+	+	+
**CYP-15-192**	TR34/L98H	4	>80	+	+	+	+	+	+	+
**CYP-15-195**	TR34/L98H	8	>80	+	+	+	+	+	+	+
**CYP-15-202**	TR34/L98H	8	>80	+	+	+	+	+	+	+
**CYP-15-212**	TR34/L98H	8	>80	+	+	+	+	+	+	+
**CYP-15-213**	TR34/L98H	4	>80	+	+	-	+	+	+	+
**CYP-15-215**	Unknown	8	>80	+	+	-	+	+	+	+
**CYP-15-220**	TR34/L98H	4	>80	+	+	+	+	+	+	+
**CYP-15-221**	TR34/L98H	8	>80	+	+	+	+	+	+	+
**CYP-15-222**	TR34/L98H	4	>80	+	+	+	+	+	+	+
**CYP-15-224**	Unknown	8	>80	+	+	+	+	+	+	+
**CYP-15-225**	Unknown	8	>80	+	+	+	+	+	+	+
**CYP-15-226**	TR34/L98H	4	>80	+	+	+	+	+	+	+
**CYP-15-228**	TR34/L98H	4	>80	+	+	+	+	+	+	+
**CYP-15-229**	TR34/L98H	8	>80	+	+	+	+	+	+	+
**CYP-15-230**	TR34/L98H	8	>80	+	+	+	+	+	+	+
**CYP-15-231**	TR34/L98H	4	>80	+	+	+	+	+	+	+
**CYP-15-75**	Unknown	8	>80	+	+	+	+	+	+	+
**CYP-15-108**	Unknown	8	>80	+	+	+	+	+	+	+
**CYP-15-109**	Unknown	>8	>80	---	---	+	---	---	---	---
**CYP-15-147**	Unknown	8	>80	+	+	+	-	-	+	+
**F14946**	Unknown	8	>80	+	+	+	+	+	+	+
**20089320**	Unknown	8	>80	+	+	+	+	+	+	+

Obs.: 1) VOR=voriconazole; BRI=Brilacidin. (+): no inhibition; (-): partial inhibition; and (---) total inhibition;

2) In the experiment, one strain (CYP15-15-109) was totally inhibited by VOR, showing Wild Type response; also, one strain (CYP15-15-147) was partially inhibited by VOR 0.5 µg/ml+BRI but the inhibition was not seen at VOR 2.0 µg/ml+BRI.

Supplementary Table S2. Protein kinase inhibitors analysed in this study

COMPOUND NAME	TARGET KINASE	PKI [20 μM]	PKI [20 μM] + BRI [20 μM]	SMILES	molecular weight (MW)
PF +3758309	PAK3	++	++	CC1=NC2=C(SC=C2)C(NC3=NNC4=C3CN(C(N[C@@H](C5=CC=CC=C5)CN(C)C)=O)C4(C)C)=N1	490.634
FRAX597		++	++	CCN1C2=NC(=NC=C2C=C(C1=O)C3=C(C=C(C=C3)C4=CN=CS4)Cl)NC5=CC=C(C=C5)N6CCN(CC6)C	558.108
FRAX486		++	+	ClC1=CC(Cl)=C(C2=CC3=CN=C(NC4=CC(F)=C(N5CCNCC5)C=C4)N=C3N(CC)C2=O)C=C1	513.403
G5555		++	++	CNC1=NC=C2C=C(C3=C(C=C(C4=CC=CC(C)=N4)C=C3)Cl)C(N(C2=N1)C[C@H]5OC[C@@H](CO5)N)=O	492.966
GW300660X	PRKAA1	++	++	NS(=O)(=O)c1ccc(N\N=C2/C(=O)Nc3ccc(cc23)C(=O)NCCc2c[nH]cn2)cc1	453.482
GW296115X		++	++	COc1ccc2[nH]c3c4[nH]c5ccc(OC)cc5c4c4C(=O)NC(=O)c4c3c2c1	385.378
GW416981X		++	++	CC(C)COC(=O)c1ccc2NC(=O)\C(=C/Nc3ccc(cc3)S(N)(=O)=O)c2c1	415.469
GW290597X		++	++	NS(=O)(=O)c1ccc(N\N=C2/C(=O)Nc3ccc(cc23)C(=O)NCCCn2ccnc2)cc1	467.509
CHIR +99021	GSK3A	++	++	N#CC(C=N1)=CC=C1NCCNC2=NC=C(C3=NC(C)=CN3)C(C4=CC=C(Cl)C=C4Cl)=N2	465.347
SB 216763		++	++	Clc1ccc(c(Cl)c1)C1=C(C(=O)NC1=O)c1cn(C)c2ccccc12	371.222
LY2090314		++	++	O=C(N1)C(C2=CN=C3N2C=CC=C3)=C(C4=CN5C6=C4C=C(F)C=C6CN(C(N7CCCCC7)=O)CC5)C1=O	512.543
AZD1080		++	++	OC(NC1=C2C=C(C#N)C=C1)=C2C3=NC=C(CN4CCOCC4)C=C3	334.378
AZD +8055	MTOR	++	++	C[C@H]1COCCN1C2=NC(=NC3=C2C=CC(=N3)C4=CC(=C(C=C4)OC)CO)N5CCOC[C@@H]5C	465.552
vistusertib		++	++	C[C@H]1COCCN1C2=NC(=NC3=C2C=CC(=N3)C4=CC(=CC=C4)C(=O)NC)N5CCOC[C@@H]5C	462.552
Torin 2		++	++	C1=CC(=CC(=C1)N2C(=O)C=CC3=CN=C4C=CC(=CC4=C32)C5=CN=C(C=C5)N)C(F)(F)F	432.404
CC +115		++	++	CCN1C(=O)CNC2=NC=C(N=C21)C3=C(N=C(C=C3)C4=NC=NN4)C	336.358
D4476	CSNK1G2	++	++	C1COC2=C(O1)C=CC(=C2)C3=C(NC(=N3)C4=CC=C(C=C4)C(=O)N)C5=CC=CC=N5	398.421
NK +258		++	++	CN(C)C(C=C1)=NN2C1=NC=C2C3=CC=CC(OC(F)(F)F)=C3	322.289
PP121	STK25	++	+	C1CCC(C1)N2C3=C(C(=N2)C4=CN=C5C(=C4)C=CN5)C(=NC=N3)N	319.371
BX +795		++	++	O=C(NCCCNC1=C(C=NC(NC2=CC=CC(NC(N3CCCC3)=O)=C2)=N1)I)C4=CC=CS4	591.472
SCH772984	MAPK3 (ERK1)	++	++	C1CN(CC1C(=O)NC2=CC3=C(C=C2)NN=C3C4=CC=NC=C4)CC(=O)N5CCN(CC5)C6=CC=C(C=C6)C7=NC=CC=N7	587.686
GW305074X		++	++	Oc1c(Br)cc(C=C2C(=O)Nc3ccc(I)cc23)cc1Br	520.942
XMD8 +92		++	++	CCOC1=C(C=CC(=C1)N2CCC(CC2)O)NC3=NC=C4C(=N3)N(C5=CC=CC=C5C(=O)N4C)C	474.563
GW300657X		++	++	NS(=O)(=O)c1ccc(N\N=C2/C(=O)Nc3ccc(cc23)C(=O)NCc2ccncc2)cc1	450.478
PF +4708671	RPS6KB2	++	++	CCC1=CN=CN=C1N2CCN(CC2)CC3=NC4=C(N3)C=C(C=C4)C(F)(F)F	390.412
LY +2584702		++	++	CC1=CC=C(C=C1)S(=O)(=O)O.CN1C=C(N=C1C2CCN(CC2)C3=NC=NC4=C3C=NN4)C5=CC(=C(C=C5)F)C(F)(F)F	617.626
AT7867		++	++	C1CNCCC1(C2=CC=C(C=C2)C3=CNN=C3)C4=CC=C(C=C4)Cl.Cl.Cl	410.775
VX +745	MAPK11	++	++	C1=CC(=C(C(=C1)Cl)C2=C3C=CC(=NN3C=NC2=O)SC4=C(C=C(C=C4)F)F)Cl	436.268
AKI00000001a		++	++	FC(C=C1)=CC(F)=C1C2=C(C(C=C3)=NN4C3=NN=C4C(O)(C)C)N(CCC5)C5=N2	396.4
AKI00000067a		++	++	FC(C=C1)=CC(F)=C1C2=C(C(C=C3)=CN4C3=NN=C4C(C)C)N(C=CO5)C5=N2	379.369
GSK223810A		++	++	COc1cc2c(Nc3cccc(NC(=O)c4ccccc4)c3)ncnc2cc1OCCCN1CCOCC1	513.596

ralimetinib		++	++	CC(C)(C)CN1C2=C(C=CC(=N2)C3=C(N=C(N3)C(C)(C)C)C4=CC=C(C=C4)F)N=C1N.CS(=O)(=O)O.CS(=O)(=O)O	612.746
TAK +715	CSNK1E	++	++	CCC1=NC(=C(S1)C2=CC(=NC=C2)NC(=O)C3=CC=CC=C3)C4=CC=CC(=C4)C	399.517
PF +670462		++	++	C1CCC(CC1)N2C=NC(=C2C3=NC(=NC=C3)N)C4=CC=C(C=C4)F.Cl.Cl	410.323
CK1 +IN +1		++	++	C1=CC=C2C(=C1)C=CC(=C2F)C3=NC(=C(N3)C4=CC=NC=C4)C5=CC=C(C=C5)F	383.4
PF +5006739		++	++	C1CN(CCC1N2C=NC(=C2C3=NC(=NC=C3)N)C4=CC=C(C=C4)F)CC5=NOC=C5	419.463
MK +8353	MAPK1	++	++	CC(C)OC1=NC=C(C=C1)C2=NNC3=C2C=C(C=C3)NC(=O)[C@@]4(CCN(C4)CC(=O)N5CCC(=CC5)C6=CC=C(C=C6)C7=NN(C=N7)C)SC	691.859
PHA +767491	CDC7	++	++	C1CNC(=O)C2=C1NC(=C2)C3=CC=NC=C3	213.239
XL413		++	++	C1C[C@H](NC1)C2=NC3=C(C(=O)N2)OC4=C3C=C(C=C4)Cl	289.721
LY3177833		++	++	C[C@@]1(C2=C(C=C(C=C2)C3=CNN=C3)C(=O)N1)C4=NC=NC=C4F	309.303
refametinib	MAP2K2	++	++	COC1=CC(=C(C(=C1NS(=O)(=O)C2(CC2)C[C@@H](CO)O)NC3=C(C=C(C=C3)I)F)F)F	572.337
selumetinib		++	++	CN1C=NC2=C1C=C(C(=C2F)NC3=C(C=C(C=C3)Br)Cl)C(=O)NOCCO	457.686
SR +3677	CAMK1G	++	++	O=C(C3COC(C=CC=C4)=C4O3)NC1=CC=C(C2=CNN=C2)C=C1OCCN(C)C.Cl.Cl	481.378
CX +4945	CSNK2A2	++	++	C1=CC(=CC(=C1)Cl)NC2=NC3=C(C=CC(=C3)C(=O)O)C4=C2C=CN=C4	349.776
SGC +CK2 +1	CSNK2A2	++	++	CC1=CC=C(NC2=NC3=C(C#N)C=NN3C(NC4CC4)=C2)C=C1NC(CC)=O	375.435
TTP 22		++	++	CC(C=C1)=CC=C1C2=CSC3=NC=NC(SCCC(O)=O)=C32	330.4
Sorafenib	MAPK13	++	++	CNC(=O)C1=NC=CC(=C1)OC2=CC=C(C=C2)NC(=O)NC3=CC(=C(C=C3)Cl)C(F)(F)F	464.83
5 +iodo tubercidin	MAP2K3	+	++	O[C@H]1[C@H](N2C=C(I)C3=C(N)N=CN=C32)O[C@H](CO)[C@H]1O	392.148
SB +216385		++	++	Nc1nccc(n1) +c1c(ncn1CCCN1CCOCC1) +c1ccc(F)cc1	382.442
SB +203580	PRKCA	++	++	CS(=O)C1=CC=C(C=C1)C2=NC(=C(N2)C3=CC=NC=C3)C4=CC=C(C=C4)F	377.442
enzastaurin		++	++	CN1C=C(C2=CC=CC=C21)C3=C(C(=O)NC3=O)C4=CN(C5=CC=CC=C54)C6CCN(CC6)CC7=CC=CC=N7	515.615
darovasertib		++	++	CC1(CCN(CC1)C2=C(N=CC=C2)NC(=O)C3=NC(=CN=C3N)C4=C(C=CC=N4)C(F)(F)F)N	472.474
GW461487A		++	++	Cl.Cc1cccc2c(c(nn12) +c1ccc(F)cc1) +c1ccncc1	339.8
UNC +ALM +33		++	++	CC1=CC2=NC(C3=CC=C(F)C=C3)=C(C4=CC=NC=C4)N2C=C1	303.339
UNC +ALM +39		+	++	CC(C=N1)=CN2C1=C(C3=CC=NC=C3)C(C4=CC=C(F)C=C4)=N2	304.327
UNC +ALM +16		+	++	CC(C=C1)=NN2C1=C(C3=CC=NC=C3)C(C4=CC=C(F)C=C4)=N2	304.327
UNC +ALM +87		++	++	FC1=CC=C(C2=C(C3=CC=NC=C3)N4C(SC(C)=C4)=N2)C=C1	309.367
GW434756X		++	++	Fc1ccc(cc1) +c1nn2ccccc2c1 +c1ccncc1	289.31

++: growth ≥ 80% compared to no treatment

+: growth ≤ 60% compared to no treatment

Supplementary Table S3. Susceptibility of phosphatase and kinase mutants to brilacidin

Strain	CAS [0.25 µg/mL]	BRI [20 µM]
**Reference strains**		
CEA17 (wild-type; A1160)	+	+
Afs35 (wild-type; A1159)	+	+
**phosphatase null mutants**		
Δ*pphA (Afu5g12010)*	+	+
Δ*sitA (Afu6g11470)*	+	+
Δ*ppzA (Afu2g03950)*	+	+
Δ*pptA (Afu5g06700)*	+	+
Δ*ptcA (Afu1g15800)*	+	+
Δ*ptcB (Afu1g09280)*	+	+
Δ*ppmA (Afu8g04580)*	+	+
Δ*ptcD (Afu5g13740)*	+	+
Δ*ptcE (Afu2g03890)*	+	+
Δ*ptcF (Afu1g06860)*	+	+
Δ*ptcG (Afu5g13340)*	+	+
Δ*ptcH (Afu4g00720)*	+	+
Δ*nemA (Afu1g09460)*	+	+
Δ*psrA (Afu1g04790)*	+	+
Δ*ppsA (Afu5g11690)*	+	+
Δ*dspD (Afu2g02760)*	+	+
Δ*cdcA (Afu3g12250)*	+	+
Δ*dspA (Afu1g13040)*	+	+
Δ*dspB (Afu1g03540)*	+	+
Δ*ptpB (Afu3g10970)*	+	+
Δ*pypA (Afu4g04710)*	+	+
Δ*ltpA (Afu2g01880)*	+	+
Δ*yphA (Afu4g07000)*	+	+
Δ*ptyA (Afu6g06650)*	+	+
Δ*calA (Afu5g09360)*	+	-
**kinases null mutants**		
Δ*mpkA*	-	-
Δ*mpkB*	+	+
Δ*mpkC*	+	+
Δ*sakA*	+	+
Δ*sakA;* Δ*mpkC*	+	+

+: sensitivity similar to wild-type

-: more sensitive than the wild-type

Supplementary Table S4. Strains used in this work		
***Aspergillus fumigatus* strains**	**Genotype**	**Reference**
CEA17 (A1160)	akuB (KU80)Δ;pyrG1 MAT1-1	1
Af293	wild-type	2
ΔpphA	ΔpphA::pyrG, akuB (KU80)Δ, MAT1-1	3
ΔsitA	ΔsitA::pyrG, akuB (KU80)Δ, MAT1-1	3
ΔppzA	ΔppzA::pyrG, akuB (KU80)Δ, MAT1-1	3
ΔpptA	ΔpptA::pyrG, akuB (KU80)Δ, MAT1-1	3
ΔptcA	ΔptcA::pyrG, akuB (KU80)Δ, MAT1-1	3
ΔptcB	ΔptcB::pyrG, akuB (KU80)Δ, MAT1-1	3
ΔppmA	ΔppmA::pyrG, akuB (KU80)Δ, MAT1-1	3
ΔptcD	ΔptcD::pyrG, akuB (KU80)Δ, MAT1-1	3
ΔptcE	ΔptcE::pyrG, akuB (KU80)Δ, MAT1-1	3
ΔptcF	ΔptcF::pyrG, akuB (KU80)Δ, MAT1-1	3
ΔptcG	ΔptcG::pyrG, akuB (KU80)Δ, MAT1-1	3
ΔptcH	ΔptcH::pyrG, akuB (KU80)Δ, MAT1-1	3
ΔnemA	ΔnemA::pyrG, akuB (KU80)Δ, MAT1-1	3
ΔpsrA	ΔpsrA::pyrG, akuB (KU80)Δ, MAT1-1	3
ΔppsA	ΔppsA::pyrG, akuB (KU80)Δ, MAT1-1	3
ΔdspD	ΔdspD::pyrG, akuB (KU80)Δ, MAT1-1	3
ΔcdcA	ΔcdcA::pyrG, akuB (KU80)Δ, MAT1-1	3
ΔdspA	ΔdspA::pyrG, akuB (KU80)Δ, MAT1-1	3
ΔdspB	ΔdspB::pyrG, akuB (KU80)Δ, MAT1-1	3
ΔptpB	ΔptpB::pyrG, akuB (KU80)Δ, MAT1-1	3
ΔpypA	ΔpypA::pyrG, akuB (KU80)Δ, MAT1-1	3
ΔltpA	ΔltpA::pyrG, akuB (KU80)Δ, MAT1-1	3
ΔyphA	ΔyphA::pyrG, akuB (KU80)Δ, MAT1-1	3
ΔptyA	ΔptyA::pyrG, akuB (KU80)Δ, MAT1-1	3
ΔcalA	ΔcalA::pyrG, akuB (KU80)Δ, MAT1-1	3
ΔmpkC	*ΔmpkC::prtA*	4
ΔsakA	*ΔsakA::hph*	4
ΔmpkA	*ΔmpkA::hph*	5
ΔmpkB	ΔakuBmpkB::ptrA; prtA+	6
ΔsakA; ΔmpkC	*ΔmpkC::prtA ΔsakA::hph*	7
Afs35 (A1159)	akuA::loxP	8
CYP-15-75	clinical isolate	9
CYP-15-108	clinical isolate	9
CYP-15-109	clinical isolate	9
CYP-15-147	clinical isolate	9
20089320	clinical isolate	9
CYP-15-184	clinical isolate	Katrien Lagrou lab
CYP-15-190	clinical isolate	Katrien Lagrou lab
CYP-15-192	clinical isolate	Katrien Lagrou lab
CYP-15-195	clinical isolate	Katrien Lagrou lab
CYP-15-202	clinical isolate	Katrien Lagrou lab
CYP-15-212	clinical isolate	Katrien Lagrou lab
CYP-15-213	clinical isolate	Katrien Lagrou lab
CYP-15-215	clinical isolate	Katrien Lagrou lab
CYP-15-220	clinical isolate	Katrien Lagrou lab
CYP-15-221	clinical isolate	Katrien Lagrou lab
CYP-15-222	clinical isolate	Katrien Lagrou lab

CYP-15-224	clinical isolate	Katrien Lagrou lab
CYP-15-225	clinical isolate	Katrien Lagrou lab
CYP-15-226	clinical isolate	Katrien Lagrou lab
CYP-15-228	clinical isolate	Katrien Lagrou lab
CYP-15-229	clinical isolate	Katrien Lagrou lab
CYP-15-230	clinical isolate	Katrien Lagrou lab
CYP-15-231	clinical isolate	Katrien Lagrou lab
F14946	clinical isolate	Gustavo Goldman lab
CM7555	clinical isolate	Gustavo Goldman lab
DPL1033	clinical isolate	David Perlin lab
MD24053	clinical isolate	David Perlin lab
***Candida spp.* strains**	**Genotype**	**Reference**
C. auris 467/2015	clinical isolate	Arnaldo Colombo lab
C. auris 468/2015	clinical isolate	Arnaldo Colombo lab
C. auris 469/2015	clinical isolate	Arnaldo Colombo lab
C. auris 470/2015	clinical isolate	Arnaldo Colombo lab
C. auris 474/2015	clinical isolate	Arnaldo Colombo lab
C. albicans DPL1006	clinical isolate; CAS^R	David Perlin lab
C. albicans DPL1007	clinical isolate; CAS^R	David Perlin lab
C. albicans DPL1009	clinical isolate; CAS^R	David Perlin lab
C. albicans DPL1010	clinical isolate; CAS^R	David Perlin lab
C. albicans DPL1011	clinical isolate; CAS^R	David Perlin lab
Cryptococcus neoformans	**Genotype**	**Reference**
H99 (ATCC 208821)	wild-type	ATCC database
**Mucorales fungi**		
R. oryzae 99-892	Lung isolate	Fungus Testing Laboratory at the University of Texas Health Sciences Center at San Antonio (UTHSCSA)
R. delemar 99-880	Brain isolate	Fungus Testing Laboratory at the University of Texas Health Sciences Center at San Antonio (UTHSCSA)
L. corymbifera	clinical isolate from DEFEAT Mucor	Ibrahim laboratory
M. circinelloides *f. jenssenii* UTHSCSA DI15-131	clinical isolate	Fungus Testing Laboratory at the University of Texas Health Sciences Center at San Antonio (UTHSCSA)

References

1. Da Silva Ferreira ME, Kress MR, Savoldi M, Goldman MH, Härtl A, Heinekamp T, Brakhage AA,Goldman GH. 2006. The akuB(KU80) mutant deficient for nonhomologous end joining is a powerful tool foranalysing pathogenicity in Aspergillus fumigatus.
2. Pain A, Woodward J, Quail MA, Anderson MJ, Clark R, Collins M, Fosker N, Fraser A, Harris D, Larke N, Murphy L, Humphray S, O'Neil S, Pertea M, Price C, Rabbinowitsch E, Rajandream MA, Salzberg S, Saunders D, Seeger K, Sharp S, Warren T, Denning DW,
3. Winkelströter LK, Dolan SK, Fernanda Dos Reis T, Bom VL, Alves de Castro P, Hagiwara D, Alowni R, Jones GW, Doyle S, Brown NA, Goldman GH. Systematic Global Analysis of Genes Encoding Protein Phosphatases in Aspergillus fumigatus. G3 (Bethesda). 2015
4. Hagiwara D., Suzuki S., Kamei K., Gonoi T., Kawamoto S. The role of AtfA and HOG MAPK pathway in stress tolerance in conidia of *Aspergillus fumigatus*. *Fungal Genet. Biol.* 2014;73:138–149.
5. Valiante V., Jain R., Heinekamp T., Brakhage A.A. The MpkA MAP kinase module regulates cell wall integrity signaling and pyomelanin formation in *Aspergillus fumigatus*. *Fungal Genet. Biol.* 2009;46(12):909–918.
6. Manfiolli AO, Siqueira FS, dos Reis TF, Van Dijck P, Schrevens S, Hoefgen S, Föge M, Straßburger M, de Assis LJ, Heinekamp T, Rocha MC, Janevska S, Brakhage AA, Malavazi I, Goldman GH, Valiante V. 2019. Mitogenactivated protein kinase cross-talk
7. Bruder Nascimento A.C., Dos Reis T.F., de Castro P.A., Hori J.I., Bom V.L., de Assis L.J., Goldman G.H. Mitogen activated protein kinases SakA (HOG1) and MpkC collaborate for *Aspergillus fumigatus* virulence. *Mol. Microbiol.* 2016;100:841–859.

8. FGSC, Fungal Genetic stock center
9. Bastos RW, Rossato L, Valero C, Lagrou K, Colombo AL and Goldman GH (2019) Potential of Gallium as an Antifungal Agent. Front. Cell. Infect. Microbiol. 9:414. doi: 10.3389/fcimb.2019.00414

Supplementary Table S5 – Primers used in this work

**Primer**	Sequences	Gene	ID
**chsA FW**	5’-CTGGAGTGTGGCTGGTCTCT-3’	*chsA*	AFUB_018960
**cshA REV**	5’-GCGTGTGAAAGCAGTATGGA-3’	*chsA*	AFUB_018960
**chsB FW**	5’-GCTCTCCACTGTCGGTCTCT-3’	*chsB*	AFUB_098840
**chsB REV**	5’-GGTCGTTGTTGATGGTGTTG-3’	*chsB*	AFUB_098840
**chsC FW**	5’-TTGCTGCGAGTTTGTATTCC-3’	*chsC*	AFUB_049230
**chsC REV**	5’-GCCAGTAGGATGCCAAAGAG-3’	*chsC*	AFUB_049230
**chsD FW**	5’-CAGAACACGATCCGAACAAC-3’	*chsD*	AFUB_012070
**chsD REV**	5’-GCTTCGCACCCAAGTAGAAC-3’	*chsD*	AFUB_012070
**chsE FW**	5’-TGGTGTTCGTTGACTTGCTC-3’	*chsE*	AFUB_029080
**chsE REV**	5’-TCATCCATCCAACCATTTCC-3’	*chsE*	AFUB_029080
**chsF FW**	5’-AACCTGCTTCTTCTGGGTGA-3’	*chsF*	AFUB_081920
**chsF REV**	5’-GAGCACGAGTTCCATGAGGT-3’	*chsF*	AFUB_081920
**chsG FW**	5’-AGGATGAGGGCAAAGAGGTT-3’	*chsG*	AFUB_034810
**chsG REV**	5’-AAGGCGTTGCTAAAGATCCA-3’	*chsG*	AFUB_034810
**csmB FW**	5’-ACAATACGCGGCGAATCC-3’	*csmB*	AFUB_029070
**csmB REV**	5’-GTTATCCCGACTGCCCAAAA-3’	*csmB*	AFUB_029070
