# Supplementary material for: A host defense peptide mimetic, brilacidin, potentiates caspofungin antifungal activity against human pathogenic fungi
Source: Nat Commun. 2023 Apr 12;14:2052. doi: 10.1038/s41467-023-37573-y (PMC10090755; doi:10.1038/s41467-023-37573-y)
Supplement: Supplementary file 3 — Reporting Summary [file 41467_2023_37573_MOESM3_ESM.pdf]

nature portfolio

Corresponding author(s): Goldman GH

Last updated by author(s): Mar 17, 2023

Reporting Summary

Nature Portfolio wishes to improve the reproducibility of the work that we publish. This form provides structure for consistency and transparency in reporting. For further information on Nature Portfolio policies, see our Editorial Policies and the Editorial Policy Checklist.

Statistics

For all statistical analyses, confirm that the following items are present in the figure legend, table legend, main text, or Methods section.

n/a	Confirmed	
☐	☒	The exact sample size (*n*) for each experimental group/condition, given as a discrete number and unit of measurement
☒	☐	A statement on whether measurements were taken from distinct samples or whether the same sample was measured repeatedly
☐	☒	The statistical test(s) used AND whether they are one- or two-sided<br>*Only common tests should be described solely by name; describe more complex techniques in the Methods section.*
☒	☐	A description of all covariates tested
☐	☒	A description of any assumptions or corrections, such as tests of normality and adjustment for multiple comparisons
☐	☒	A full description of the statistical parameters including central tendency (e.g. means) or other basic estimates (e.g. regression coefficient) AND variation (e.g. standard deviation) or associated estimates of uncertainty (e.g. confidence intervals)
☐	☒	For null hypothesis testing, the test statistic (e.g. *F*, *t*, *r*) with confidence intervals, effect sizes, degrees of freedom and *P* value noted<br>*Give P values as exact values whenever suitable.*
☒	☐	For Bayesian analysis, information on the choice of priors and Markov chain Monte Carlo settings
☒	☐	For hierarchical and complex designs, identification of the appropriate level for tests and full reporting of outcomes
☐	☒	Estimates of effect sizes (e.g. Cohen's *d*, Pearson's *r*), indicating how they were calculated

Our web collection on statistics for biologists contains articles on many of the points above.

Software and code

Policy information about availability of computer code

Data collection	No software was used
Data analysis	No software was used

For manuscripts utilizing custom algorithms or software that are central to the research but not yet described in published literature, software must be made available to editors and reviewers. We strongly encourage code deposition in a community repository (e.g. GitHub). See the Nature Portfolio guidelines for submitting code & software for further information.

Data

Policy information about availability of data

All manuscripts must include a data availability statement. This statement should provide the following information, where applicable:

- Accession codes, unique identifiers, or web links for publicly available datasets
- A description of any restrictions on data availability
- For clinical datasets or third party data, please ensure that the statement adheres to our policy

Data availability statement. All the data are available as supplementary tables and figures. Drug compounds were obtained from the Pandemic Response box (400 compounds) and COVID box (160 compounds) (both available at https://www.mmv.org), the National Institutes of Health (NIH) clinical collection (727 compounds) (available at https://pubchem.ncbi.nlm.nih.gov/source/ NIH%20Clinical%20Collection) and the SGC's epigenetic chemical probe and SGC's pharma donated chemical probe libraries (115 compounds, see https://www.thesgc.org/chemical-probes) totaling 1,402 compounds. Source data are provided with this paper.

Human research participants

Policy information about studies involving human research participants and Sex and Gender in Research.

Reporting on sex and gender	Our current studies do not involve human research
Population characteristics	Our current studies do not involve human research
Recruitment	Our current studies do not involve human research
Ethics oversight	Our current studies do not involve human research

Note that full information on the approval of the study protocol must also be provided in the manuscript.

Field-specific reporting

Please select the one below that is the best fit for your research. If you are not sure, read the appropriate sections before making your selection.

☒ Life sciences ☐ Behavioural & social sciences ☐ Ecological, evolutionary & environmental sciences

For a reference copy of the document with all sections, see nature.com/documents/nr-reporting-summary-flat.pdf

Life sciences study design

All studies must disclose on these points even when the disclosure is negative.

Sample size	The sample size used in this study was usually determined based on the cost, time, or convenience of collecting the data, and the need for it to offer sufficient statistical power. The rationale for determining the sample size is based on routine experiments where at least three samples are used for microbial or growth or metabolic activity while sample size for cell lines and animal experiments was between 6 and 10 samples.
Data exclusions	No data were excluded from the analysis
Replication	All the experiments in this manuscript were repeated at least twice and most of the time, three times. All attempts of replication were successful
Randomization	All the experiments were randomized either by their position in the incubator chambers for microbial growth and metabolic activity, or in the distribution of cages in the cage incubator
Blinding	Although all the experiments were randomized, blinding was not relevant for interpreting the results of our study. We are not dealing with clinical trials or experiments that have to be blinded.

Reporting for specific materials, systems and methods

We require information from authors about some types of materials, experimental systems and methods used in many studies. Here, indicate whether each material, system or method listed is relevant to your study. If you are not sure if a list item applies to your research, read the appropriate section before selecting a response.

Materials & experimental systems

n/a	Involved in the study	
☒	☐	Antibodies
☐	☒	Eukaryotic cell lines
☒	☐	Palaeontology and archaeology
☐	☒	Animals and other organisms
☒	☐	Clinical data
☒	☐	Dual use research of concern

Methods

n/a	Involved in the study	
☒	☐	ChIP-seq
☒	☐	Flow cytometry
☒	☐	MRI-based neuroimaging

Eukaryotic cell lines

Policy information about cell lines and Sex and Gender in Research

Cell line source(s)	A549 (Banco de Células do Rio de Janeiro, https://bcrj.org.br/)
Authentication	The cell line used was acquired and authenticated by the cell bank in Rio de Janeiro, Brazil (https://bcrj.org.br/)
Mycoplasma contamination	The cell line was tested for mycoplasm contamination by using the methodology described by Young L, Sung J, Stacey G, Masters JR. Detection of Mycoplasma in cell cultures. Nat Protoc. 2010;5:929–34. The mycoplasm contamination was verified by a PCR test.
Commonly misidentified lines (See ICLAC register)	There are no misidentified cell lines in our study. Our the cell line tested negative for mycoplasma contamination.

Animals and other research organisms

Policy information about studies involving animals; ARRIVE guidelines recommended for reporting animal research, and Sex and Gender in Research

Laboratory animals	Mice BALB/c and C57BL/6, six to eight weeks old. Cages are well ventilated, softly lit and subjected to a 12:12 light-dark cycle. The relative humidity was kept at 40 to 60%. Mouse rooms and cages were kept at a temperature range of 22oC.
Wild animals	This study did not involve wild animals
Reporting on sex	Sex was visually assigned. Females for BALB/c and males for C57BL/6
Field-collected samples	The study did not involve samples collected from the field
Ethics oversight	The principles that guide our studies are based on the Declaration of Animal Rights ratified by UNESCO on January 27, 1978 in its 8th and 14th articles. All protocols adopted in this study were approved by the local ethics committee for animal experiments from the University of São Paulo, Campus of Ribeirão Preto (Permit Number: 08.1.1277.53.6; Studies on the interaction of Aspergillus fumigatus with animals). Groups of five animals were housed in individually ventilated cages and were cared for in strict accordance with the principles outlined by the Brazilian College of Animal Experimentation (COBEA) and Guiding Principles for Research Involving Animals and Human Beings, American Physiological Society. All efforts were made to minimize suffering. Animals were clinically monitored at least twice daily and humanely sacrificed if moribund (defined by lethargy, dyspnea, hypothermia and weight loss). All stressed animals were sacrificed by cervical dislocation.

Note that full information on the approval of the study protocol must also be provided in the manuscript.
